# Association of the gut microbiota with clinical variables in obese and lean Emirati subjects

**DOI:** 10.3389/fmicb.2023.1182460

**Published:** 2023-08-23

**Authors:** Manal Ali Ahmad, Mirey Karavetian, Carole Ayoub Moubareck, Gabi Wazz, Tarek Mahdy, Koen Venema

**Affiliations:** ^1^School of Nutrition and Translational Research in Metabolism (NUTRIM), Faculty of Health, Medicine and Life Sciences, Maastricht University, Maastricht, Netherlands; ^2^Faculty of Kinesiology and Physical Education, University of Toronto, Toronto, ON, Canada; ^3^College of Natural and Health Sciences, Zayed University, Dubai, United Arab Emirates; ^4^Center of Excellence in Bariatric and Metabolic Surgery, Dr. Sulaiman Al Habib Hospital, Dubai, United Arab Emirates; ^5^Department of Bariatric Surgery, Sharjah University, Sharjah, United Arab Emirates; ^6^Centre for Healthy Eating and Food Innovation (HEFI), Maastricht University-Campus Venlo, Venlo, Netherlands

**Keywords:** gut microbiota, obesity, firmicutes, bacteroidetes, 16S rRNA, United Arab Emirates

## Abstract

**Background:**

Growing evidence supports the role of gut microbiota in obesity, yet exact associations remain largely unknown. Specifically, very little is known about this association in the Emirati population.

**Methods:**

We explored differences in gut microbiota composition, particularly the Firmicutes/Bacteroidetes (F/B) ratio, between 43 obese and 31 lean adult Emirate counterparts, and its association with obesity markers, by using V3-V4 regions of 16 S ribosomal RNA gene sequencing data. Furthermore, we collected anthropometric and biochemical data.

**Results:**

The two major phyla in obese and lean groups were Firmicutes and Bacteroidetes. We observed a significantly lower alpha diversity (Shannon index) in obese subjects and a significant difference in beta diversity and phylum and genus levels between the two groups. The obese group had higher abundances of Verrucomicrobia and Saccharibacteira and lower abundances of Lentisphaerae. *Acidaminococcus* and *Lachnospira* were more abundant in obese subjects and positively correlated with adiposity markers. No correlations were found between the gut microbiota and biochemical variables, such as fasting blood sugar, total cholesterol, HDL cholesterol, LDL cholesterol, and triglycerides.

**Conclusion:**

We reveal significant differences in the gut microbiota between obese and lean adult Emiratis and an association between certain microbial genera of the gut microbiota and obesity. A better understanding of the interactions between gut microbes, diet, lifestyle, and health is warranted.

## 1. Introduction

Obesity is a multifactorial disease (Cannon and Kumar, 2009), declared by the World Health Organization (WHO) in 2016 as a pandemic (World Health Organization, 2016). Long-standing factors involved in obesity include excessive caloric intake and a sedentary lifestyle (Morgen and Sørensen, 2014). More recently, obesity was also found to present a complicated disequilibrium of the diversity, richness, and evenness of the bacterial component of the gut microbiota (Aguirre and Venema, 2015), known as dysbiosis (Adlerberth and Wold, 2009; Round and Mazmanian, 2009). The latter has been proposed as part of the etiology of obesity (Davis, 2016) through its effect on digestion, regulation of metabolism, adipose tissue composition, short-chain fatty acid (SCFA) production, modulation of the production of gut peptides and hormones, among others (Corfe et al., 2015; Patrone et al., 2016). The human gut microbiota is primarily composed of Firmicutes and Bacteroidetes, which make up about 90% of all bacterial species, in addition to Actinobacteria, Fusobacteria, Proteobacteria, and Verrucomicrobia (Abenavoli et al., 2019; Rinninella et al., 2019). Nevertheless, substantial differences in composition and function in the microbiota between obese and healthy individuals have been established (Turnbaugh and Gordon, 2009; Tremaroli and Bäckhed, 2012; Pinart et al., 2021). In some studies, obesity in humans is typically characterized by high intestinal concentrations of Firmicutes and low concentrations of Bacteroidetes (Seganfredo et al., 2017; Crovesy et al., 2020); whereby the higher Firmicutes-to-Bacteroidetes ratio (F/B) among obese (Armougom et al., 2009; Furet et al., 2010; Kong et al., 2013) is potentially associated with a greater energy harvest from host diet (Turnbaugh et al., 2006). However, other studies show the opposite F/B ratio in obesity (Schwiertz et al., 2010), or no relation at all (Duncan et al., 2008) and thus, the cause-and-effect relationship between F/B and obesity is still to be elucidated.

Improvements in bacterial DNA sequencing allowed researchers to understand the gut microbiota and its composition and explore its complex relationship with health and disease (Gordon et al., 2007; Duncan et al., 2008). However, there are still gaps in our understanding of the role of the gut microbiota in the etiology of obesity and the effectiveness of interventions targeting gut microbiota for preventing and managing obesity (Tehrani et al., 2012), “e.g., it is still not entirely clear whether differences in the microbiota cause obesity, or whether obesity causes a difference in microbiota composition.” Primarily, this is because most studies have used rodent models, which have different gut microbiota composition, fermentation process, and dietary practices than humans (Heinritz et al., 2013; Nguyen et al., 2015). Moreover, inconsistencies in human studies might be attributed to the different approaches to analyzing the composition of the gut microbiota, as well as the recruitment of subjects with ethnic differences and inter-individual dissimilarities in genetics, diets, and lifestyles which can shape the composition of the gut microbiota (Seganfredo et al., 2017; Crovesy et al., 2020; Pinart et al., 2021; Zeng et al., 2021). The characteristics of the gut microbiota in subjects with obesity are highlighted. Nevertheless, little is known about the association between gut microbiota and clinical variables (fasting blood sugar, total cholesterol, HDL cholesterol, LDL cholesterol, triglycerides, and anthropometrics) linked to obesity in humans. This warrants scientific investigations to explore microbial patterns associated with obesity markers.

In the Gulf countries, the prevalence of obesity is steadily increasing. Specifically, in the United Arab Emirates (UAE), the prevalence of overweight and obesity doubled between 1989 and 2017 (Radwan et al., 2018), and there are 27.8% of adults obese, according to the National Health Survey 2017–2018 (Sulaiman et al., 2017; Ministry of Health and Prevention, 2023). In this work, we aimed to profile the gut microbiota and its association with clinical variables and explore differences (through a comparative analysis) between obese and lean Emirate subjects. Exploring the gut microbiome of Emiratis adds a piece of the puzzle to our understanding of obesity, with the ultimate goal of uncovering preventive and interventional measures tailored to the Emirati population aiming to curb the obesity epidemic in the country.

## 2. Materials and methods

### 2.1. Study design

This is a cross-sectional study conducted among Emirati subjects living in the UAE. The present study is part of a pre-post study registered at clinicaltrials.gov (NCT04200521) and performed in the UAE.

### 2.2. Ethical considerations

Ethical approval was granted by the Research Ethics Committee of the Ministry of Health and Prevention of UAE (MOHP/DXB-REC-52/2018), the Dubai Health Care Regulatory Research Ethics Committee (DHCR-REC), and the Zayed University Ethical Committee Board (ZU19_51_F). The study followed the Declaration of Helsinki. Written informed consent was collected from all study subjects.

### 2.3. Participants and sample size

We determined the sample size by following standard calculations based on normal distributions. The sample size was calculated based on the change in F/B from pre- to post-bariatric surgery (Brant, 2015), based on the study by Damms-Machado et al. (2015). In that study, the mean(SD) fecal F/B increased significantly from 5.9 (2.1) to 10.4 (1.4) in 3 months post-bariatric surgery (Damms-Machado et al., 2015). To detect a similarly significant effect, two patients were needed to achieve 80% power at a two-sided alpha level of 5%. To allow for the high expected dropout rate, the minimum sample size was multiplied by 15. Hence, we targeted 30 Emirati obese adults residing in the UAE of both sex and planning to undergo the bariatric procedure. In addition, we recruited a sample of 30 age and sex-matched control subjects (lean adults living in the UAE).

### 2.4. Inclusion criteria

Emirati residents of UAE, aged between 18 and 60 years, of either sex, free of antibiotics for at least 3 months, falling into one of the two body mass index (BMI) categories were included: (1) obese with a BMI of ≥35 kg/m^2^ and willing to undergo bariatric surgery; (2) lean counterparts with a BMI of 18.5–24.9 kg/m^2^ and consenting to participate in the study.

Obese participants were recruited from one hospital in Dubai and another in Sharjah. In contrast, lean subjects were recruited from community settings in Dubai through word-of-mouth and flyer postings. Recruitment took place from October 2019 till March 2021.

### 2.5. Exclusion criteria

Individuals who consumed alcohol exceeding two drinks per day for men and one drink per day for women (Snetselaar et al., 2021), were pregnant at the time of the study, experienced significant weight loss (≥5%) in the past 3 months, or were not willing to consent for the study were excluded.

### 2.6. Outcome measures and data collection

General demographic characteristics collected from the patient included age and sex. Furthermore, health-related information was collected using a subjective screening questionnaire, including the presence of any chronic diseases such as dyslipidemia, diabetes, and hypertension, surgical history in the past 5 years, weight loss of at least 5% in the past 3 months, current intake of medication including antibiotics, and current consumption of prebiotic/probiotic/fiber supplement and probiotic foods, consumption of other nutrition supplements, and engagement in a physically active lifestyle and exercise.

Physical activity level was analyzed using two questions from our initial screening questionnaire. A “yes” or “no” answer was required for the following two questions: “Do you integrate physical activity in your daily routine” and “Do you incorporate any exercise program.” Sedentary was denoted if “no” was answered to both questions; light physical activity if “yes” was only answered to the first question; moderate physical activity if “yes” was only responded to the second question; and high physical activity if “yes” was answered to both questions.

### 2.7. Anthropometric and clinical variables

For each participant, anthropometric measurements, lipid profile, fasting blood sugar (FBS), and microbiota analyses were done.

*Anthropometric data* were collected by trained research assistants and dietitians.

Body weight (kg): measured via portable Seca 762 scale (Vogel & Halke, Hamburg, Germany), following best practices, i.e., with light clothes and without shoes. Height (cm): measured using a portable stadiometer attached to the weighing scale following best practices, i.e., to the nearest 0.1 cm, without shoes, with the participant stretching to the maximum height while having the head positioned in the Frankfort plane. BMI (kg/m^2^): calculated according to the standard formula by dividing weight in kilograms by squared height in meters. Waist circumference (WC) (cm): measured using a Seca 201 ergonomic circumference measuring tape, at the mid-point between the right iliac crest and the lower costal region, to the nearest 0.1 cm (Van der Kooy and Seidell, 1993). WC was categorized as appropriate (<94 cm in men and < 80 cm in women) or increased ( ≥ 94 cm for men and ≥ 80 cm for women; Ford, 2005).

Waist-to-height ratio (WHtR): calculated by dividing the WC (cm) by height (cm), whereby abdominal obesity was defined as a WHtR ≥ 0.5 (Ashwell and Gibson, 2016). Fat mass (FM) and Percent Body Fat (PBF): measured via bioelectrical impedance analyzer (BC-420 MA, Tanita Corporation, Tokyo, Japan) with the participants being well hydrated, without drinking caffeine for 12 h, not participating in excessive physical activity 24 h before the analysis, and wearing comfortable clothes. Elevated PBF was defined as PBF ≥ 25% and ≥ 35% in men and women, respectively (Romero-Corral et al., 2008).

### 2.8. Biochemical parameters

Total cholesterol (TC), low-density lipoprotein cholesterol (LDL-C), high-density lipoprotein cholesterol (HDL-C), triglycerides (TG), and FBS: measured after 12 h of fasting via portable Lux Meter Blood Test (Biochemical Systems International, S.p.A; Arezzo, Italy) following the manufacturer’s instructions.

### 2.9. Stool sample collection

Stool samples were collected in Zymo DNA/RNA Shield fecal collection tubes and stored at room temperature. Two samples were collected from each participant, one of which was analyzed, and the second was held for accuracy verification. Sample collection followed standard protocols and regulations. Collection tubes (with a spoon attached to the cap for collecting 1 gram of feces) were pre-filled with DNA/RNA Shield™ (9 mL). The nucleic acids (DNA & RNA) in the samples are preserved at room temperature (DNA > 1 year, RNA up to 1 month).

### 2.10. DNA isolation and sequencing of the V3–V4 region of the 16S rRNA gene

DNA isolation and sequencing of the barcoded amplicons of the V3–V4 region of the 16S rRNA gene was done as per the protocols of Illumina (Illumina, Eindhoven, The Netherlands; Surono et al., 2022). The sequencing was performed using the Illumina MiSeq system (San Diego, CA, United States) using barcodes and the 2 × 300 bp protocol. We used Quantitative Insights Into Microbial Ecology: QIIME 2^1^ software to analyze raw sequences; the latter were classified using the Silva database (version 132) as a reference 16S rRNA gene database. Reads were filtered, and only those present in at least 20% of the samples were taken.

#### 2.10.1. Diversity analysis

*α Diversity indices*: observed-OTUs, Chao1 index, Phylogenetic diversity (Faith’s PD), Pielou evenness, and Shannon diversity index, which were calculated with QIIME 2 and shown using the R software [R (4.1.x) (R Core Team)]^2^ in RStudio. The rarefaction curves were generated using the ggplot2 package for R. Rarefication depth was set at 3800.

*β Diversity* indices were visualized in a Principal Coordinate Analysis (PCoA) based on Bray–Curtis dissimilarity, Jaccard distance, and weighted and unweighted UniFrac using QIIME2 software.

The statistical significance of β diversity differences between the two groups was determined using Permutational Multivariate Analysis of Variance (PERMANOVA). The value of p was calculated through the Pairwise permanova method to compare β diversity between each category in all samples. Differences between obesity and lean samples (at phyla and genus levels) were analyzed via the non-parametric Kruskal–Wallis test corrected with the Benjamini–Hochberg false discovery rate (FDR) for multiple comparisons in Rstudio (the software package R (4.1.x), R Core Team, footnote 2).

The composition of the two groups was visualized using the Krona tool.^3^

Linear discriminant analysis (LDA) effect size (LEfSe) was used to explore microbial taxa with differential abundance between lean and obese (version 1.0).^4^ Taxa showing LDA values above two at a value of *p* <0.05 were considered enriched taxa in each group.

The non-parametric Spearman’s rank-order correlations between the Amplicon Sequence Variant (ASVs) and continuous variables (BMI, WC, WHtR, PBF, FM, LDL, HDL, TC, TG, TC/HDL, and FBS) were calculated. *Q*-values, i.e., adjusted value of *p*s after the FDR, were considered significantly different at a cut-off of <0.05. The F/B ratio was calculated by dividing the relative abundances of Firmicutes and Bacteroidetes.

A heatmap of Spearman correlation analysis was generated in R software (ggplot2 package).

### 2.11. Statistical analyses

We analyzed ‘participants’ characteristics using the Statistical Package for the Social Sciences software version 21 (SPSS Inc. Chicago, IL, United States). We assessed the normality of the data using Skewness and Kurtosis. Normal data were summarized as mean (M) ± standard deviation (SD) and skewed data as median (Mdn) and interquartile range (IQR). Between-group differences were explored using independent samples t-test for normally distributed continuous variables, Mann–Whitney U-test for skewed continuous variables, and Chi-square for categorical values. A value of *p* < 0.05 was used to denote statistical significance.

## 3. Results

The characteristics of participant groups are presented in Table 1. Forty-three obese subjects were included (37.2% men; mean ± SD age of 29.95 ± 9.13 years), whereas 31 lean subjects participated (38.7% men; mean ± SD age 29.67 ± 10.73 years). No significant differences in gender and age were observed between the groups. The obese group had higher anthropometric (BMI, WC, WHtR, and PBF) and biochemical parameters (FBS, LDL, TC, and TC/HDL-c ratio) than the lean group, as expected. There was no significant difference in lifestyle factors between the two groups (prebiotic, probiotic, fiber, alcohol consumption, and smoking). The level of physical activity in the obese group was mainly light and significantly lower than that of the lean group (Supplementary Table S1).

**Table 1:** Demographic, anthropometric, and biochemical parameters between lean (*n* = 31) and obese participants (*n* = 43).

	Lean [mean ± SD or *n* (%)]	Obese [mean ± SD or n (%)]	Value of *p*
Age (years)	29.67 ± 10.73	29.95 ± 9.13	0.565
Female	19 (61.3)	27 (62.8)	0.896
Weight (kg)	62.39 ± 8.17	118.23 ± 25.6	<0.001*
Height (cm)	166.29 ± 8.38	165.16 ± 9.77	0.603
BMI (kg/m^2^)	22.49 ± 1.93	43.12 ± 6.83	<0.001*
WC (cm)	76.70 ± 14.83	123.29 ± 17.76	<0.001*
Appropriate	23 (74.2)	0 (0)	<0.001*
Increased risk	8 (25.8)	43 (100)	
WtHR	0.48 ± 0.05	0.74 ± 0.08	<0.001*
Normal	16 (51.6)	0 (0)	<0.001*
High	15 (48.4)	43 (100)	
PBF (%)	26.62 ± 9.39	46.13 ± 30	<0.001*
FM (kg)	17.13 ± 7.12	53.8 ± 13.3	<0.001*
MM (kg)	43.14 ± 9.02	57.43 ± 11.76	<0.001*
FFM in (kg)	45.91 ± 9.27	62.08 ± 11.83	<0.001*
FFMI (FFM kg/m^2^)	16.46 ± 2.22	22.77 ± 2.72	<0.001*
FBS (mg/dL) Median (IQR)	92 (87–98)	97 (93.6–104)	0.003*
HDL (mg/dL)	43.63 ± 15.78	41.74 ± 11.05	0.547
LDL (mg/dl)	73.93 ± 20.99	98.65 ± 32.16	<0.001*
TG (mg/dL)	123.24 ± 35.49	128.53 ± 62.07	0.645
TC (mg/dL)	138 (120–159)	161.6 (143–181.7)	0.001*
TC/HDL ratio	3.54 ± 1.10	4.18 ± 1.22	0.022*

* Denotes statistical significance (*p* < 0.05) between groups. Independent-sample *t*-test was used for normally distributed continuous variables with mean, and SD (Age, BMI, WC, WtHR, PBF, FM, MM, FFM, FFMI, LDL, HDL, TG, TC/HDL-c), Mann–Whitney U-test for skewed continuous variables (FBS, TC) with IQR and medians, and Chi-square test was used for categorical variables. BMI, body mass index; WC, waist circumference; PBF, percentage body fat; FM, fat mass; WtHR, waist-to-height ratio; MM, muscle mass; FFM, fat-free mass; FFMI, fat-free mass index; FBS, fasting blood sugar; HDL, high- density lipoprotein cholesterol; LDL, low-density lipoprotein cholesterol; TG, triglycerides; TC, total cholesterol; TC/HDL, total cholesterol to high-density lipoprotein cholesterol ratio.

### 3.1. Differences in bacterial composition between the obese and lean groups

After filtering for taxa that were present in at least 20% of the samples, the microbiota was composed of eight phyla and 109 genera.

At the phylum level, Firmicutes (50% in lean vs. 47% in obese) and Bacteroidetes (44% in lean vs. 49% in obese) were the two most abundant bacterial phyla in the gut of obese and lean subjects. The obese group had significantly higher abundances of the phyla Verrucomicrobia (0.5% in obese vs. 0.3% in lean, Benjamini-Hochberg correction, *q* = 0.048), and Saccharibacteira (0.003% in obese vs. 0.0009% in lean, *q* = 0.0002) than the lean group and significantly lower abundances of the phyla Lentisphaerae (0.03% in obese vs. 0.08% in lean, *q* = 0.004). However, there was no significant difference in the relative abundance of Bacteroidetes (*q* = 0.12), Firmicutes (*q* = 0.32), Fusobacteria (*q* = 0.2), Actinobacteria (*q* = 0.54), and Proteobacteria (*q* = 0.85) among the two groups. Furthermore, there was no significant difference in the F/B ratio between the obese (0.95) and lean group (1.1); *p* = 0.315 (Supplementary Figures S1A,B; Table 2).

**Table 2:** The gut microbial composition (relative abundance in %) of obese and lean participants (phylum level).

Phylum	Lean (%)	Obese (%)	*q* *
Firmicutes	50	47	0.32
Bacteroidetes	44	49	0.12
Proteobacteria	4	3	0.85
Actinobacteria	0.6	0.5	0.54
Verrucomicrobia	0.3	0.5	0.04*
Lentisphaerae	0.08	0.03	0.004*
Fusobacteria	0.0003	0.0001	0.20
Saccharibacteria	0.0009	0.003	0.0002*
F/B ratio	1.1	0.95	0.315

* *q* ≤ 0.05 was considered statistically significant.

### 3.2. Genus level

Taxonomic assignment was also performed at the genus level and revealed a total of 21 major genera with a significant difference between the obese and lean group, where 16 of these belonged to Firmicutes, as depicted in Table 3.

**Table 3:** The difference in bacterial composition (relative abundance in %) between obese and lean subjects (genus level).

Genus	Lean (%)	Obese (%)	q value
**Higher in lean subjects**			
*Clostridiales.vadinBB60.group.D_5-uncultured. Bacterium*	0.1	0.01	0.001*
*Anaerotruncus*	0.3	0.1	0.001*
*Ruminococcaceae.D_5__uncultured*	0.7	0.2	0.003*
*Ruminococcaceae.UCG.014*	1	0.4	0.004*
*Ruminococcaceae.UCG.010*	0.4	0.05	0.008*
*Family.XIII.UCG.001*	0.03	0.01	0.006*
*Erysipelotrichaceae.D_5__uncultured*	0.2	0.02	0.007*
*Christensenellaceae.R.7.group*	2	0.3	0.008*
*Rhodospirillaceae.D_5__uncultured*	0.5	0.03	0.001*
*Victivallis*	0.07	0.01	0.002*
*Alistipes*	3	2	0.04*
*Lachnospiraceae.UCG.010*	0.2	0.1	0.013*
*Ruminococcaceae.UCG.005*	1	0.6	0.013*
*Ruminococcaceae.UCG.002*	2	1	0.02*
*Ruminiclostridium.6*	0.5	01	0.02*
*Lachnospiraceae.D_5__uncultured*	0.2	0.1	0.02*
*Odoribacter*	0.4	0.2	0.03*
*Barnesiella*	0.6	0.2	0.03*
*Lachnospiraceae.NK4A136.group*	1	0.9	0.04*
**Increased in obese peoples**			
*Acidaminococcus*	0.1	0.7	0.01*
*Lachnospira*	0.9	2	0.04*

* *q* ≤ 0.05 was considered statistically significant.

**Figure 1:**
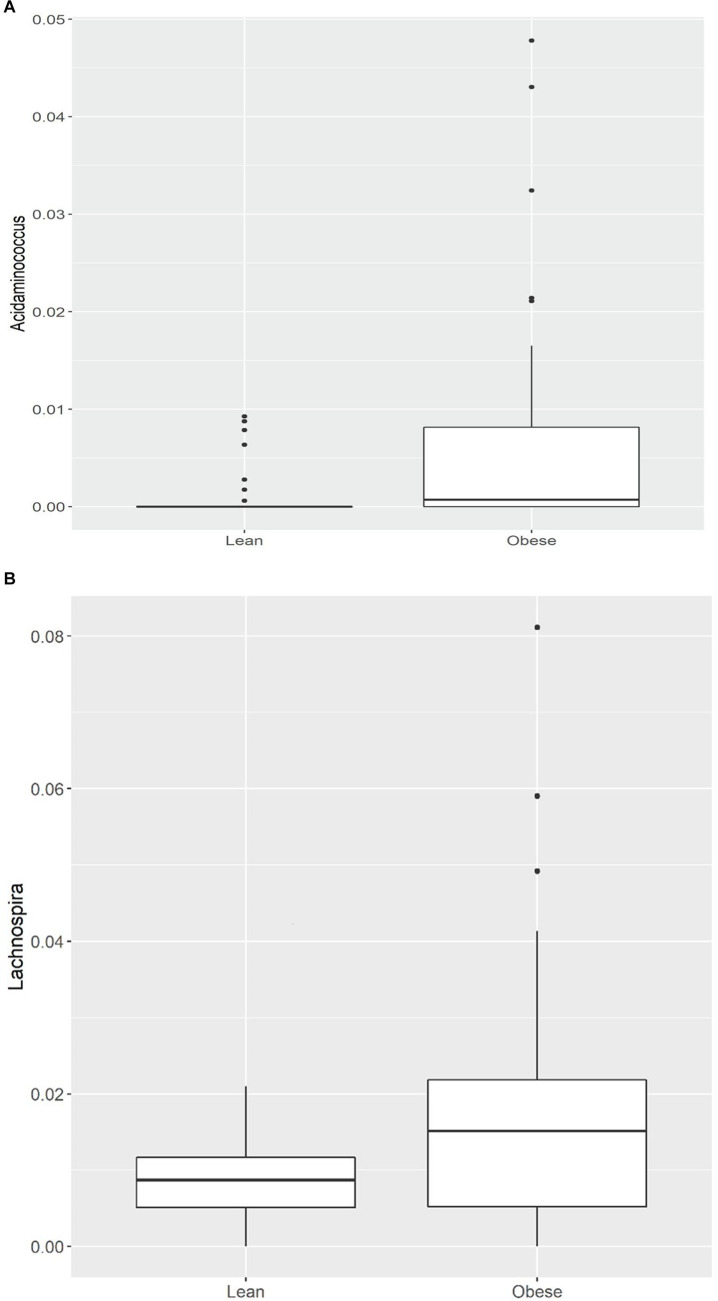
**(A)** Comparison of relative abundance of Acidaminococcus (genus level); **(B)** Comparison of relative abundance of Lachnospira (genus level) between lean and obese participants. *q < 0.05.

Of the 21 genera, *Acidaminococcus* and *Lachnospira* (belonging to the Firmicutes phylum) were higher in the obese group, while the remaining 19 were lower, including (a) from the Firmicutes phylum, an uncharacterized taxon in the Clostridiales.vadinBB60.group (*q* = 0.001), *Anaerotruncus* (*q* = 0.001), an uncharacterized Ruminococcaceae (*q* = 0.003), *Ruminococcaceae*.UCG.014 (*q* = 0.004), *Ruminococcaceae*.UCG.010 (*q* = 0.008), Family.XIII.UCG.001 (of the Clostridiales order, Family XIII; *q* = 0.006), an uncharacterized Erysipelotrichaceae (*q* = 0.007), *Christensenellaceae*.R.7.group (*q* = 0.008), *Lachnospiraceae*.UCG.010 (*q* = 0.013), *Ruminococcaceae*.UCG.005 (*q* = 0.013), *Ruminococcaceae*.UCG.002 (*q* = 0.02), *Ruminiclostridium*.6 (*q* = 0.02), an uncharacterized Lachnospiraceae (*q* = 0.02), *Lachnospiraceae*.NK4A136.group (*q* = 0.04), and (b) from the Bacteroidetes phylum, *Alistipes* (*q* = 0.04), *Odoribacter* (*q* = 0.03), *Barnesiella* (*q* = 0.03), (c) from the Proteobacteria phylum, an uncharacterized Rhodospirillaceae (*q* = 0.001), and (d) from the Verrucomicrobia phylum, *Victivallis* (*q* = 0.002; Table 3; Figures 1A,B; Supplementary Figures 2A,B).

**Figure 2:**
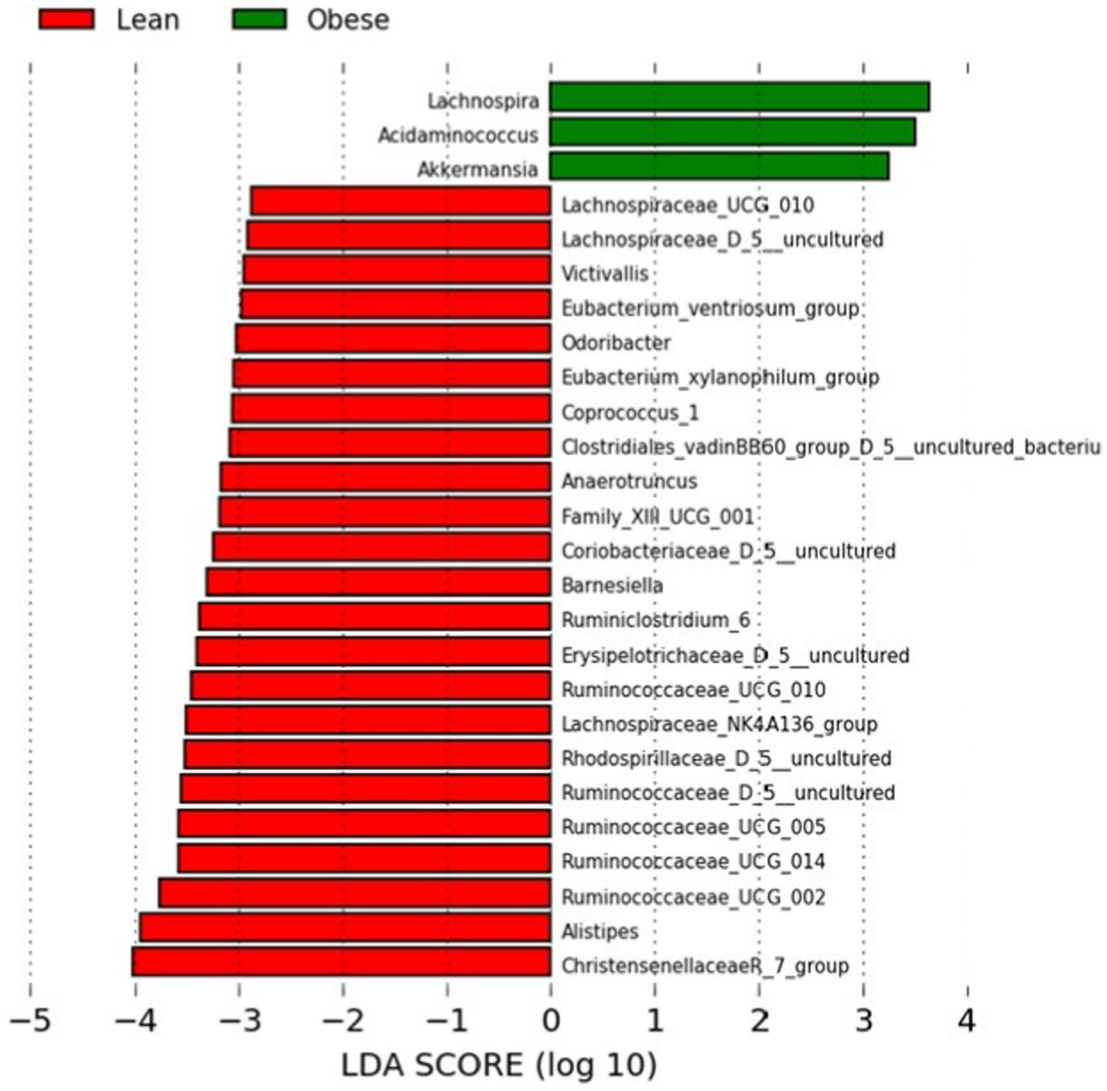
LEfSe analysis between the obese and lean groups (LDA score > 2). The LDA score (log10) for the more prevalent genera in the obese group is represented on a positive scale (green), and the LDA score for the more prevalent genera in the lean group is defined on a negative scale (red).

We performed a Linear Discriminant Analysis Effect Size (LEfSe) on the taxa from the obese and lean groups. A total of 26 genera showed significantly different abundances between the two groups with LDA scores >2. In this case, 23 genera were higher in the lean group, and three genera were higher in the obese group, as shown in Figure 2.

Of the three genera in the obese group, two belonged to Firmicutes (*Lachnospira* and *Acidaminococcus*) and one to Verrucomicrobia (*Akkermansia*). In the Kruskal-Wallis test, the latter was not statistically significant. In contrast, 17, 3, one, one, and one of the 23 genera for the lean group belonged to Firmicutes, Bacteroidetes, Actinobacteria, and Verrucumictobia phyla.

### 3.3. α- and β-Diversity (microbial diversity and richness, microbial dissimilarities)

**Figure 3:**
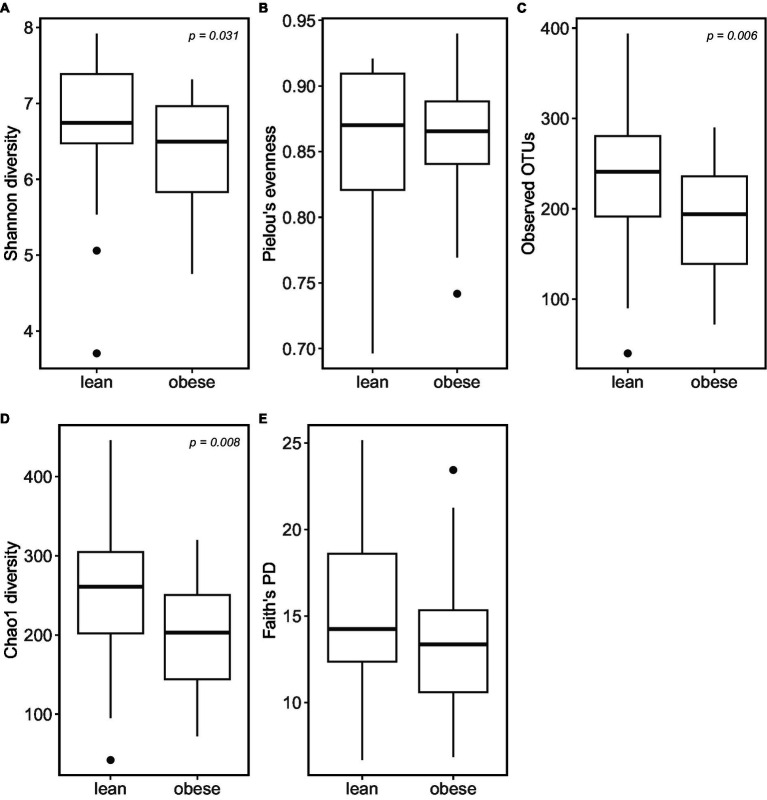
Obese individuals tend to have lower microbiome α-diversity. α-diversity was calculated using: **(A)** Shannon diversity; **(B)** Pielou’s Evenness; **(C)** Observed_OTUs; **(D)** Choa1 diversity; **(E)** Faith’s PD.

According to the α -diversity metrics (Figure 3), obese adults showed significantly lower diversity (Shannon index, *p* = 0.031), coinciding with the lower observed OTUs (*p* = 0.006) and lower Chao1 richness estimator (*p* = 0.008). Furthermore, the obese group showed lower “Faith’s phylogenetic diversity (PD; *p* = 0.069) and lower evenness (‘Pielou’s evenness, *p* = 0.53). However, the latter two were not statistically significant.

**Figure 4:**
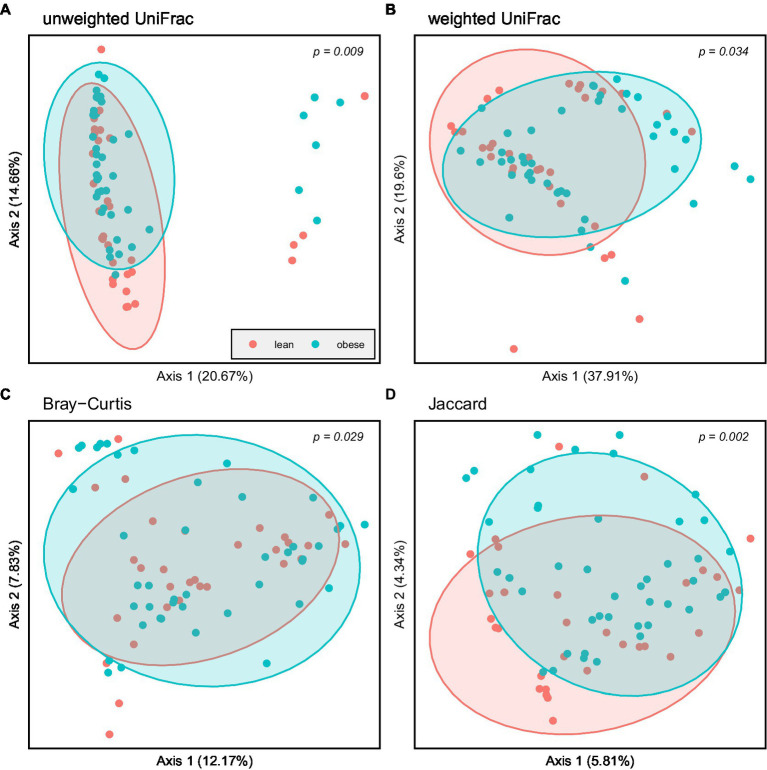
Principal coordinate Analysis (PCoA) based on: **(A)** Unweighted UniFrac; **(B)** Weighted UniFrac; **(C)** Bray–Curtis and **(D)** Jaccard; Obese (blue spheres) and lean participants (red spheres).

Principal coordinate Analysis (PCoA) based on unweighted UniFrac, weighted UniFrac, Bray–Curtis, and Jaccard showed a marked separation between the gut microbiota community of obese and lean groups, confirmed by the pairwise permanova analysis that indicated a significant difference in β-diversity between the two groups (Figure 4; *p* = 0.009; *p* = 0.034; *p* = 0.029; *p* = 0.002, respectively).

### 3.4. Correlation between gut microbiota (at genus level) and clinical variables

**Figure 5:**
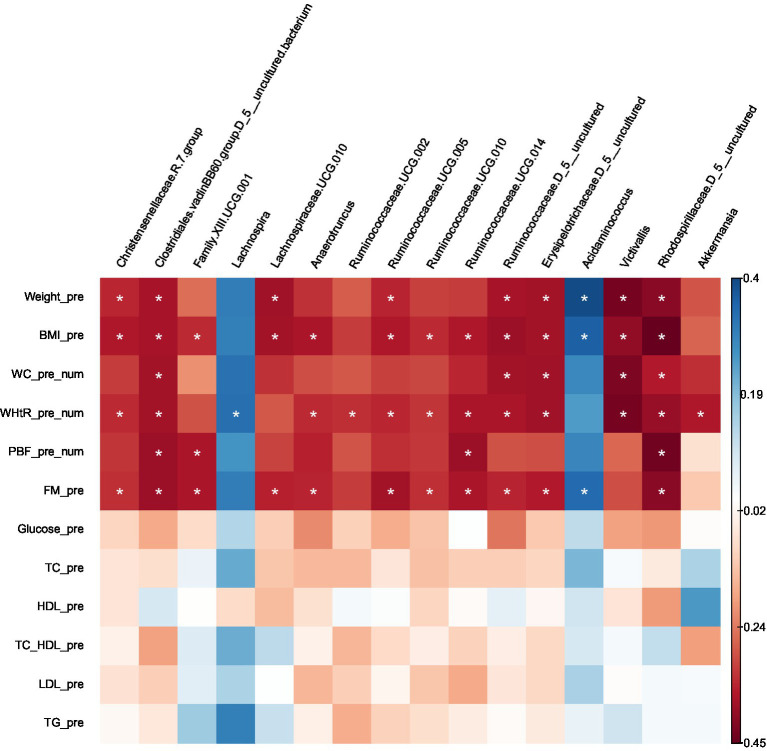
Heatmap of Spearman correlation analysis between gut microbiota (at genus level) and clinical variables.

Certain microbes were positively or negatively correlated with BMI, WC, WHtR, PBF, and FM. Statistically significant correlations between microbes and anthropometric measurements are provided in (Supplementary Table S2) and the heat map (Figure 5).

Several taxa that were significantly lower in obese compared with lean using Kruskal–Wallis or LEfSe were negatively correlated with BMI, WC, WHtR, PBF, and FM (uncharacterized Rhodospirillaceae), (uncharacterized Clostridiales.vadinBB60.group), BMI, WHtR (*Victivallis*), BMI, WC and FM (uncharacterized Ruminococcaceae), BMI, WC, WHtR and FM (uncharacterized Erysipelotrichaceae), BMI & FM (*Lachnospiraceae*.UCG.010), BMI, WHtR and FM (*Christensenellaceae*.R.7.group, *Ruminococcaceae*.UCG.005, *Ruminococcaceae*.UCG.010, *Anaerotruncus*), BMI, WHtR, PBF and FM (Ruminococcaceae.UCG.014); BMI, PBF and FM (Family.XIII.UCG.001), WHtR (*Ruminococcaceae*.UCG.002). On the other hand, the taxa which were higher in the obese group presented positive correlations with BMI and FM (*Acidaminococcus*), and WHtR (*Lachnospira*); except for *Akkermansia,* which was prevalent in the obese group (LEfSe) was found to be negatively correlated with WHtR.

No correlations were found between the gut microbiota at the genus level and the following clinical variables: FBS, total cholesterol, HDL cholesterol, LDL cholesterol, and triglycerides. Furthermore, There were no correlations with age or gender.

## 4. Discussion

Recent research on the human gut microbiota has revealed its various roles in health and disease, with obesity being among the many conditions to which changes in the gut microbiota have been linked (Jinatham et al., 2018). Research on gut microbiota and obesity has mainly focused on Western populations, with limited studies on Arabs and almost none on Emiratis. This study pioneered in contrasting the fecal microbial composition of obese and lean Emirati people using 16S rRNA gene sequencing (V3-V4 region) and exploring its association with clinical parameters. It is difficult to compare this with previous studies in Western populations, because also diet, which differs between the Arabic and Western culture, also significantly affects microbiota composition.

To the best of our knowledge, the present cross-sectional study is first to provide insights into the relationships between clinical variables and the gut microbiota in obese and lean Emirati subjects. The α- diversity richness was significantly lower in the obese than the lean Emirate without significant difference in evenness. These data are in line with the majority of available studies (Pinart et al., 2021). Conversely, β-diversity, denoting dissimilarities in gut microbiota composition, showed significant differences between obese and lean; this also agrees with most available studies. Thus, the microbiota in the obese group presented fewer bacterial taxa, and lean samples showed a more diverse gut microbiota profile than obese samples based on Shannon index results. Controversy regarding relative abundance at genus and phylum level in individuals with obesity, compared with lean subjects, was observed.

Moreover, higher genera in the obese group correlated positively with BMI and FM (*Acidaminococcus*) and WHtR (*Lachnospira*). The exception was *Akkermansia* which was more prevalent in the obese group but was negatively associated with WHtR. This is in contrast to other studies (Gao X. et al., 2018; Jinatham et al., 2018), in different populations. On the other hand, genera higher in the lean group correlated negatively with adiposity (BMI, WC, WHtR, and FM).

The structure, function, and diversity of the gut microbiota in subjects with obesity differ from those of lean people (Cheng et al., 2019). In general, people with obesity show lower richness and biodiversity (Turnbaugh et al., 2009; Cotillard et al., 2013; Le Chatelier et al., 2013; Isolauri, 2017) similar to our findings, while Kocełak et al. (2013) and Kasai et al. (2015) found greater diversity richness in individuals with obesity. Le Chatelier et al. reported that subjects with obesity who have low bacterial richness present more overall adiposity, dyslipidemia, insulin resistance, and higher inflammation compared with obese individuals who have an elevated gut bacterial richness (Le Chatelier et al., 2013; Crovesy et al., 2020). In line with the literature, the present study revealed that Firmicutes and Bacteroidetes were the two most abundant bacterial phyla in the gut of obese and lean subjects without significant differences noted at the level of Firmicutes, Bacteroidetes (Pinart et al., 2021) nor F/B ratio (Castaner et al., 2018; Jinatham et al., 2018) between the two groups. It is well known that Firmicutes and Bacteroidetes predominate the gut microbiome globally (Castaner et al., 2018). Scarce data on the gut microbiota of people in the Arabian Peninsula describe the dominance of Firmicutes and Actinobacteria as phyla in individuals from Saudi Arabia and the preponderance of either Firmicutes or Bacteroidetes in individuals from Kuwait (Plummer et al., 2020).

Of interest, it was believed in early reports that the ratio of Firmicutes and Bacteroidetes differs between lean and obese people, with a high ratio observed in the US (Plummer et al., 2020). However, this was later refuted, and the majority of evidence does not show significant differences in the relative abundance of Firmicutes (Pinart et al., 2021), whereas contradictory findings in the relative abundance of Bacteroidetes persist (Crovesy et al., 2020; Pinart et al., 2021).

The usefulness of a high F/B ratio as an indicator of obesity is still debatable and more complicated than a mere difference in the relative abundance between phyla (Turnbaugh and Gordon, 2009; Venema, 2010; Jinatham et al., 2018). The critical impact of some bacteria on human metabolism may be due to the number of less abundant bacteria or the higher abundance of other ones (Sonnenburg and Backhed, 2016). Gut microbiota geared towards the production of SCFA, rather than a simple increase in F/B ratio, may be associated with an increased energy harvest from ‘one’s diet leading to obesity. Investigating the association between energy harvest related to bacterial SCFA production and obesity is warranted (Venema, 2010; Jinatham et al., 2018). Furthermore, the F/B ratio may be strongly associated with metabolic markers of obesity, such as insulin resistance and metabolic syndrome, instead of obesity (Louis et al., 2016). Based on our results, a high F/B ratio may not be a valid global biomarker of obesity (Houtman et al., 2022). It is more likely that differences at the genus (and even species) level, compared with the phylum level, could be related to changes in metabolic function (Bauer et al., 2016). While significantly higher Lentisphaerae in lean individuals and Saccharibacteria and Verrucomicrobia in the obese group were reported, no differences in the relative abundance of Fusobacteria, Actinobacteria, and Proteobacteria are observed. The issue of differences between phyla in obese and lean individuals remains controversial in the literature (Pinart et al., 2021).

Similarly, differences between the obese and lean groups at the genus level remain controversial (Crovesy et al., 2020). Interestingly, in our study, the genus Lachnospiraceae NK4A136 was significantly higher in the lean group. Studies in mice suggest that this genus has a protective and anti-inflammatory effect being a potential butyrate producer (Companys et al., 2021). Furthermore, Companys et al. (2021) first identified the genus Lachnospiraceae NK4A136 as a biomarker of leanness in humans. Furthermore, in our study, as shown elsewhere (Gao R. et al., 2018), butyrate-producing Ruminococcus was higher in the lean group, denoting a more diverse microbiome profile.

Moreover, Christensenellaceae.group.7 (Firmicutes phylum) was significantly higher in the lean group. It is usually associated with a low BMI (Castaner et al., 2018), and when transplanted to mice, it promotes a lean host phenotype and positively impacts the diversity of the community (Aguirre and Venema, 2015). The abundance of *Alistipes* was higher in the lean group; this new genus is significantly elevated in high-fat-diet-fed mice than in controls (Yang et al., 2022); individuals with obesity have a higher abundance of *Alistipes* (Crovesy et al., 2020). This aberrant finding may be related to the fact that Emirate adults could have a distinct gut microbiota composition given their varying dietary factors and lifestyle. In UAE, there is a transition towards energy-dense diets high in animal protein, total and saturated fatty acids, and simple sugars (Cheikh Ismail et al., 2020). High-fat diets are linked to considerable changes in the gut microbiota composition at the phylum and genus levels (Rosenbaum et al., 2015).

Our finding of a negative association between the genus *Lachnospiraceae* and PBF and BMI is also reported by Lippert et al. in older adults in Austria (Lippert et al., 2017), but not in a study with human stool from Ghanaian volunteers (Dugas et al., 2018). The negative correlation between bacterial taxa that were dominant in the lean group and the adiposity markers (BMI, WC, WHtR, FM, PBF) is unexpected, considering that most of these taxa belong to the Firmicutes phylum.

In contrast, genera that were higher in obese subjects positively correlated with BMI and FM (*Acidaminococcus*) as well as WHtR (*Lachnospira*), suggesting them as potential microbiota biomarkers of obesity (Companys et al., 2021). In addition, levels of *Acidamonicoccus* positively correlated with type 2 diabetes in the Saudi population (Al-Muhanna et al., 2022). Contrary to our results, a study in overweight adults showed that reduced *Lachnospira* abundance was associated with weight gain (Companys et al., 2021). These conflicting findings may have been attained because the two genera comprise various species whose role in obesity has not been thoroughly studied; such issues are still debatable (Aguirre and Venema, 2015).

Some of the identified microbial taxa correlated with adiposity but not other biochemical parameters, i.e., glucose and lipid homeostasis markers. It is tempting to assume that taxa showing negative correlations with adiposity are appropriate markers of metabolic health. Nevertheless, larger studies are required to ascertain such relationships (Palmas et al., 2021).

The presence of divergent results between this study and other studies in the literature might be attributed to the geographical location of the studies, given dietary and environmental variations known to affect gut microbiota (Al-Muhanna et al., 2022) or to many other factors, such as the population under study, gender, physical activity, drugs, ethnicity, and the season, as well as methods used to analyze the gut microbiota, such as different DNA extraction techniques, different areas of the 16S rRNA gene sequenced and primers used. The composition of the gut microbiota can be easily influenced, and all factors that affect it are difficult to control (Crovesy et al., 2020). The majority of available studies did not evaluate factors that can affect the gut microbiota, such as diet, physical activity, smoking, stress, and sleep, which might explain the possible differences found between studies (Rosenbaum et al., 2015; Aljazairy et al., 2022). Amongst these, the diet is likely to greatly impact the gut microbiota composition (Crovesy et al., 2020).

### 4.1. Strengths and limitations

This study provides meaningful insight into the relationship between gut microbiota and obesity, specifically in Emirati adults, and is a useful addition to the international microbiome reference dataset. Yet, this study is limited by its relatively small sample size. Furthermore, we did not adjust for a potential confounding factor such as dietary intake, hindering our ability to link unique gut microbiota profiles with specific diets. Finally, our results should be interpreted cautiously as they are based on observational data.

## 5. Conclusion

We revealed significant differences in microbiota between obese and lean subjects at phylum and genus levels. Remarkably, our study showed no significant differences in Firmicutes and Bacteroidetes or F/B ratio, suggesting that F/B cannot be used as a universal predictor biomarker for obesity in adults. Genera that were higher in obese subjects correlated positively with BMI and FM (Acidaminococcus) as well as WHtR (Lachnospira) and are genera that might serve as microbiota biomarkers of obesity in Emerati. In contrast, genera higher in the lean group correlated negatively with adiposity. Future studies are needed to confirm our results and to understand better the interplay between diet, lifestyle, gut microbiota, and health. Whether changes in the gut microbial composition are a cause or a consequence of obesity remains an open question.

## Data availability statement

The raw sequences and corresponding metadata have been archived in the Sequence Read Archive (SRA) repository at the NCBI under accession number PRJNA962831: http://www.ncbi.nlm.nih.gov/bioproject/962831.

## Ethics statement

The studies involving human participants were reviewed and approved by Ethical approval was granted by the Research Ethics Committee of the Ministry of Health and Prevention of UAE (MOHP/DXB-REC-52/2018), the Dubai Health Care Regulatory Research Ethics Committee (DHCR-REC), and the Zayed University Ethical Committee Board (ZU19_51_F). The study followed the Declaration of Helsinki. Written informed consent was collected from all study subjects. The patients/participants provided their written informed consent to participate in this study. The studies were conducted in accordance with the local legislation and institutional requirements. The participants provided their written informed consent to participate in this study.

## Author contributions

MA, MK, and KV: conceptualization, methodology, and data curation. MA and KV: software and supervision. MK, KV, and CM: validation. MA: formal analysis, investigation, and writing – original draft preparation. MK, KV, CM, GW, and TM: resources and writing – review and editing. MA, MK, KV, CM, GW, and TM: visualization. MK and KV: project administration. CA and KV: funding acquisition. All authors contributed to the article and approved the submitted version.

## Funding

This research was funded by Zayed University, Dubai, UAE (grant number R19054) and the Dutch Province of Limburg with a grant to the Centre for Healthy Eating and Food Innovation (HEFI) of Maastricht University – Campus Venlo (grant number HEFI-1).

## Conflict of interest

The authors declare that the research was conducted without any commercial or financial relationships that could be construed as a potential conflict of interest.

## Publisher’s note

All claims expressed in this article are solely those of the authors and do not necessarily represent those of their affiliated organizations, or those of the publisher, the editors and the reviewers. Any product that may be evaluated in this article, or claim that may be made by its manufacturer, is not guaranteed or endorsed by the publisher.

## Footnotes

^1^https://qiime2.org/

^2^http://www.R-project.org

^3^https://github.com/marbl/Krona/wiki/KronaTools

^4^http://huttenhower.sph.harvard.edu/galaxy/
