# Supplementary material for: Association of the gut microbiota with clinical variables in obese and lean Emirati subjects
Source: Front Microbiol. 2023 Aug 23;14:1182460. doi: 10.3389/fmicb.2023.1182460 (PMC10481963; doi:10.3389/fmicb.2023.1182460)
Supplement: Supplementary file 1 [file Data_Sheet_1.docx]

Supplementary Material

Association of the gut microbiota with clinical variables in Obese and Lean Emirati subjects

**Manal Ali Ahmad ^1^, Mirey Karavetian ^2^, Carole Ayoub Moubareck ^3^, Gabi Wazz ^4^, Tarek Mahdy ^5^, KoenVenema ^6,*^**

*** Correspondence:** Corresponding Author: [k.venema@maastrichtuniversity.nl](mailto:k.venema@maastrichtuniversity.nl)

**Supplementary Table 1. Lifestyle factors for obese and lean participants**

| **Lifestyle factors** | **Lean (n=31)** | **Obese (n=43)** | **p-value** |
| --- | --- | --- | --- |
| Probiotic Supplement - Yes, n (%) | 1 (3.2) | 0 (0) | 0.236 |
| Fiber Supplement - Yes, n (%) | 0 (0) | 2 (4.7) | 0.223 |
| Probiotic Food Consumption - Yes, n (%) | 24 (77.4) | 29 (67.4) | 0.348 |
| Probiotic Food Consumption Frequency- n (%) |  |  | 0.138 |
| None | 5 (16.1) | 15 (34.9) |  |
| 2 or less/per week | 16 (51.6) | 13 (30.2) |  |
| 3 to 6/week | 9 (29) | 11 (25.6) |  |
| 7 or more/week | 1 (3.2) | 4 (9.3) |  |
| Prebiotic Supplement - Yes, n (%) | 1 (3.2) | 0 (0) | 0.236 |
| Smoker - Yes, n (%) | 3 (9.7) | 12 (27.9) | 0.054 |
| Alcohol consumption - Yes, n (%) | 0 (0) | 2 (4.7) | 0.223 |
| Physical activity level |  |  | <0.001* |
| Sedentary | 0 (0) | 6 (14) |  |
| Light | 12 (38.7) | 31 (72.1) |  |
| Moderate | 1 (3.2) | 2 (4.7) |  |
| High | 18 (58.1) | 4 (9.3) |  |

**Supplementary Table 2. Spearman correlation coefficient (rho) for the taxa showing significant correlations with clinical data**

| **BMI** | | | **WC** | | **WHtR** | | **PBF** | | **FM** | |
| --- | --- | --- | --- | --- | --- | --- | --- | --- | --- | --- |
| Genus | **rho** | **rho** | | **rho** | | **rho** | | **rho** | |  |
| uncharacterized *Rhodospirillaceae* | -0.44 | -0.35 | | -0.38 | | -0.43 | | -0.40 | |  |
| *Victivallis* | -0.39 | -0.42 | | -0.42 | |  | |  | |  |
| uncharacterized *Ruminococcaceae* | -0.38 | -0.37 | | -0.36 | |  | | -0.34 | |  |
| uncharacterized *Erysipelotrichaceae* | -0.37 | -0.37 | | -0.38 | |  | | -0.35 | |  |
| *Lachnospiraceae*.UCG.010 | -0.37 | - | |  | |  | | -0.34 | |  |
| uncharacterized Clostridiales.vadinBB60.group | -0.36 | -0.37 | | -0.37 | | -0.38 | | -0.38 | |  |
| *Anaerotruncus* | -0.36 | - | | -0.33 | | - | | -0.34 | |  |
| *Acidaminococcus* | 0.36 | - | |  | | - | | 0.34 | |  |
| *Christensenellaceae.R.7.group* | -0.36 | - | | -0.33 | | - | | -0.33 | |  |
| *Ruminococcaceae.UCG.005* | -0.35 | - | | -0.33 | | - | | -0.37 | |  |
| *Ruminococcaceae.UCG.014* | -0.35 | - | | -0.36 | | -0.38 | | -0.36 | |  |
| *Family.XIII.UCG.001* | -0.33 | - | |  | | -0.36 | | -0.36 | |  |
| *Ruminococcaceae.UCG.010* | -0.33 | - | | -0.31 | | - | | -0.32 | |  |
| *Ruminococcaceae.UCG.002* | - | - | | -0.32 | | - | | - | |  |
| *Akkermansia* | - | - | | -0.36 | | - | | - | |  |
| *Lachnospira* | - | - | | 0.34 | | - | | - | |  |


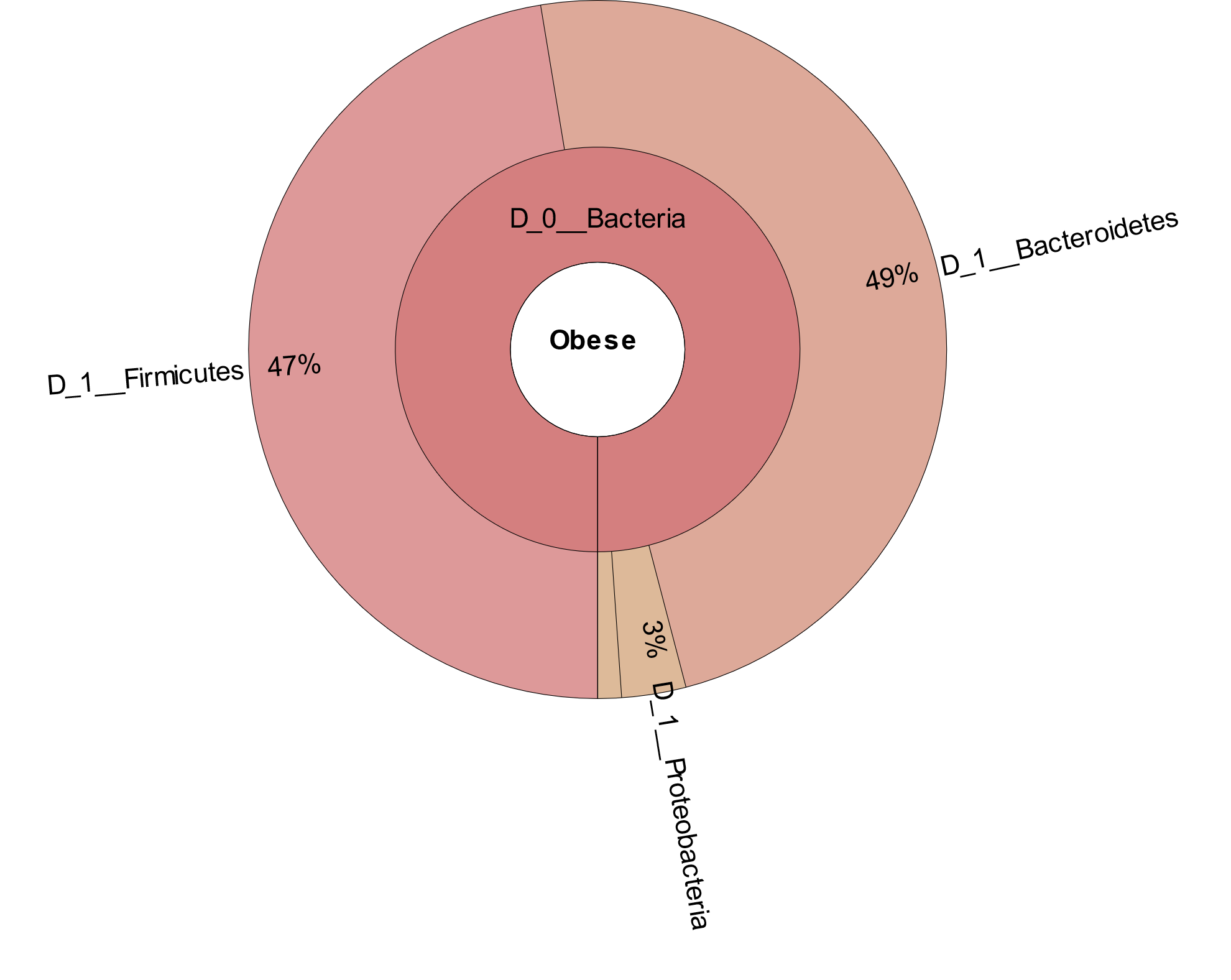
A


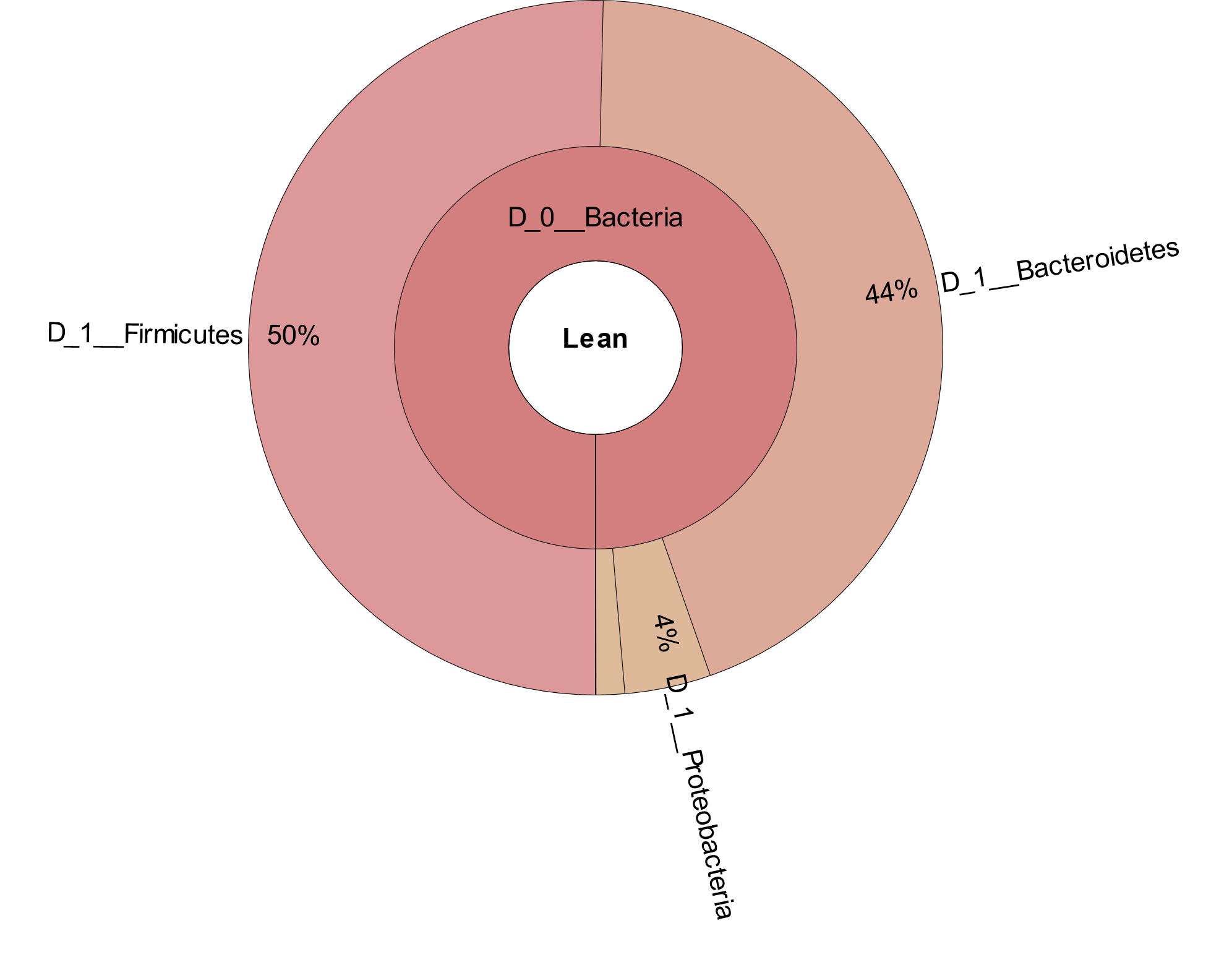
B

**Supplementary Figure 1: (A ) Average phylum distribution of gut microbiota of obese participants. (B) Average phylum distribution of gut microbiota of lean participants.**


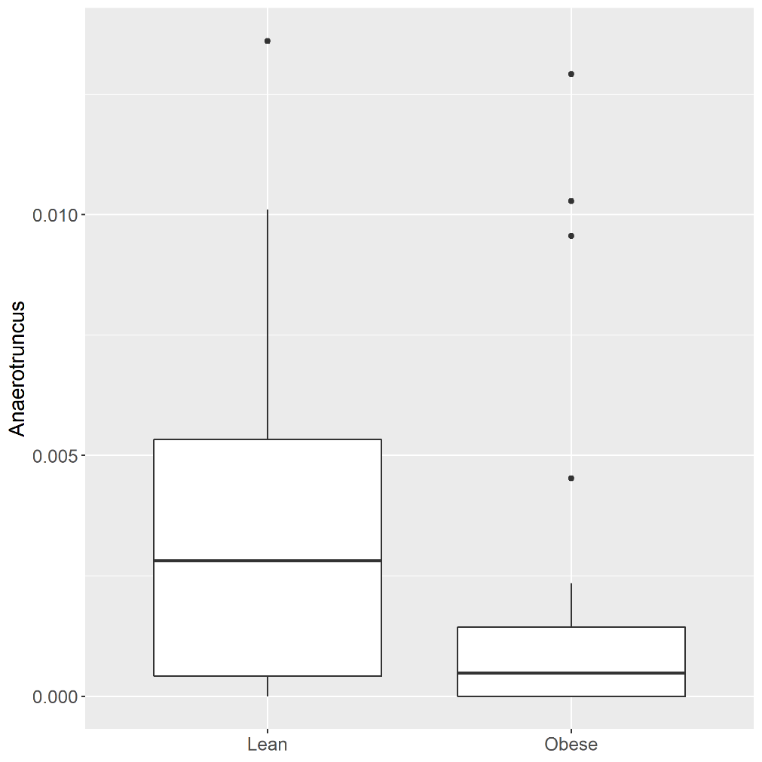
-A


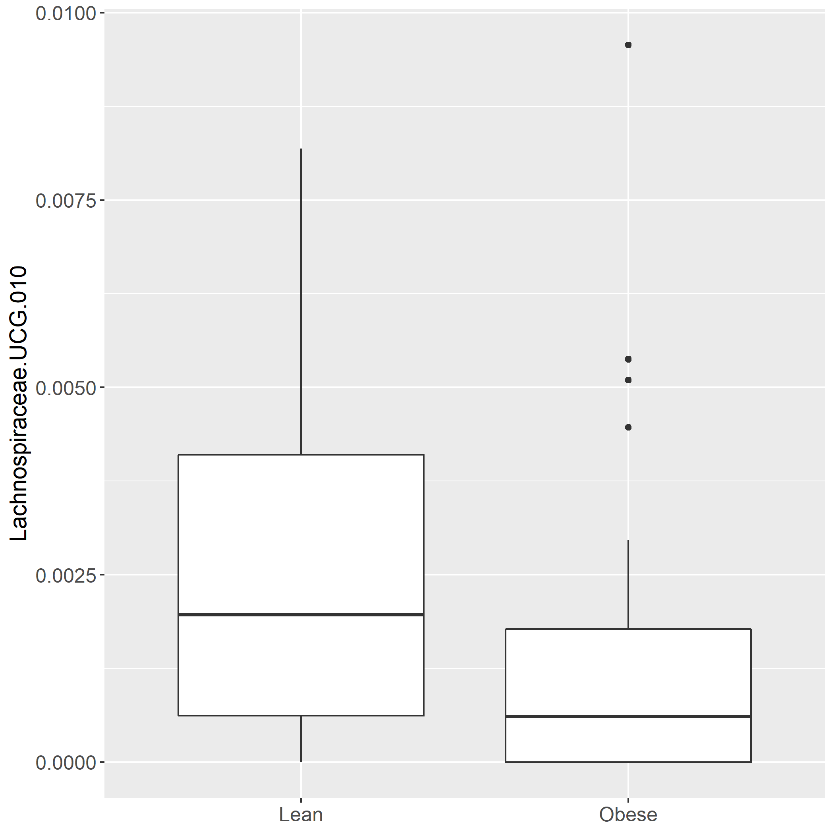
-B

**Supplementary Figure 2. (A) Comparison of relative abundance of Anaerotruncus (genus level); (B)Comparison of relative abundance of Lachnospiraceae.UCG.010 (genus level) between lean and obese participants. *q < 0.05**
